# USAID Associated with Myeloid Neoplasm and VEXAS Syndrome: Two Differential Diagnoses of Suspected Adult Onset Still’s Disease in Elderly Patients

**DOI:** 10.3390/jcm10235586

**Published:** 2021-11-27

**Authors:** Marion Delplanque, Achille Aouba, Pierre Hirsch, Pierre Fenaux, Julie Graveleau, Florent Malard, Damien Roos-Weil, Nabil Belfeki, Louis Drevon, Artem Oganesyan, Matthieu Groh, Matthieu Mahévas, Jerome Razanamahery, Gwenola Maigne, Matthieu Décamp, Sébastien Miranda, Thomas Quemeneur, Julien Rossignol, Laurent Sailler, Marie Sébert, Louis Terriou, Anna Sevoyan, Yervand Hakobyan, Sophie Georgin-Lavialle, Arsène Mekinian

**Affiliations:** 1 Service de Médecine Interne, Centre de Référence des Maladies Autoinflammatoires et des Amyloses (CEREMAIA), AP-HP, Hôpital Tenon, Sorbonne Université, 75020 Paris, France; marion.delplanque@outlook.com (M.D.); sophie.georgin-lavialle@aphp.fr (S.G.-L.); 2 Service de Médecine Interne, CHU de Caen, Hôpital de la Côte de Nacre, 14033 Caen, France; aouba-a@chu-caen.fr (A.A.); maigne-g@chu-caen.fr (G.M.); 3 Service d’Hématologie Biologique, INSERM, Centre de Recherche Saint-Antoine, AP-HP, Hôpital Saint-Antoine, Sorbonne Université, 75012 Paris, France; pierre.hirsch@aphp.fr (P.H.); florent.malard@aphp.fr (F.M.); louis.drevon@aphp.fr (L.D.); 4 Service d’Hématologie Seniors, AP-HP, Hôpital Saint-Louis, 75010 Paris, France; pierre.fenaux@aphp.fr (P.F.); marie.sebert@aphp.fr (M.S.); 5 Service de Médecine Interne, Centre Hospitalier Georges Charpak, 44600 Saint Nazaire, France; julie.graveleau@chu-nantes.fr; 6 Service d’Hématologie, AP-HP, Hôpital Pitié Salpêtrière, 75013 Paris, France; damien.roos-weil@aphp.fr; 7 Service de Médecine Interne, Centre Hospitalier Marc Jacquet, 77000 Melun, France; nabil.belfeki@ghsif.fr; 8 Department of Hematology and Transfusion Medicine, National Institute of Health, Yerevan 0051, Armenia; a.t.oganesyan@gmail.com (A.O.); sevoyananna6@gmail.com (A.S.); yero75@yahoo.com (Y.H.); 9 Service de Médecine Interne, Hôpital Foch, 92150 Suresnes, France; m.groh@hopital-foch.com; 10 Service de Médecine Interne, CHU Hôpital Henri Mondor, 94000 Créteil, France; matthieu.mahevas@aphp.fr; 11 Service de Médecine Interne, CHRU Jean Minjoz, 25000 Besançon, France; jerome.razanamahery@hotmail.fr; 12 Laboratoire de Génétique CHU de Caen, Hôpital de la Côte de Nacre, 14000 Care, France; decamp-m@chu-caen.fr; 13 Service de Médecine Interne, CHU Hôpital Charles Nicolle, 76000 Rouen, France; sebastien.miranda@chu-rouen.fr; 14 Service de Médecine Interne, CH de Valenciennes, 59300 Valenciennes, France; quemeneur-t@ch-valenciennes.fr; 15 Service d’Hématologie Adultes, AP-HP, Hôpital Necker-Enfants Malades, 75015 Paris, France; julien.rossignol@aphp.fr; 16 Service de Médecine Interne, CHU Hôpital Purpan, 31059 Toulouse, France; sailler.l@chu-toulouse.fr; 17 Service de Médecine Interne, CHR Lille, Sorbonne Université, 75005 Paris, France; louis.terriou@chru-lille.fr; 18 Service de Médecine Interne, AP-HP, Hôpital Saint Antoine, Sorbonne Université, 75012 Paris, France

**Keywords:** adult-onset Still’s disease, myelodysplastic syndrome, SAID, USAID, VEXAS, azacytidine

## Abstract

Background: Patients with solid cancers and hematopoietic malignancy can experience systemic symptoms compatible with adult-onset Still’s disease (AOSD). The newly described VEXAS, associated with somatic UBA1 mutations, exhibits an overlap of clinical and/or biological pictures with auto inflammatory signs and myelodysplastic syndrome (MDS). Objectives: To describe a cohort of patients with signs of undifferentiated systemic autoinflammatory disorder (USAID) concordant with AOSD and MDS/chronic myelomonocytic leukemia (CMML) and the prevalence of VEXAS proposed management and outcome. Methods: A French multicenter retrospective study from the MINHEMON study group also used for other published works with the support of multidisciplinary and complementary networks of physicians and a control group of 104 MDS/CMML. Results: Twenty-six patients were included with a median age at first signs of USAID of 70.5 years with male predominance (4:1). Five patients met the criteria for confirmed AOSD. The most frequent subtypes were MDS with a blast excess (31%) and MDS with multilineage dysplasia (18%). Seven patients presented with acute myeloid leukemia and twelve died during a median follow-up of 2.5 years. Six out of 18 tested patients displayed a somatic UBA1 mutation concordant with VEXAS, including one woman. High-dose corticosteroids led to a response in 13/16 cases and targeted biological therapy alone or in association in 10/12 patients (anakinra, tocilizumab, and infliximab). Azacytidine resulted in complete or partial response in systemic symptoms for 10/12 (83%) patients including 3 VEXAS. Conclusions: Systemic form of VEXAS syndrome can mimic AOSD. The suspicion of USAID or AOSD in older males with atypia should prompt an evaluation of underlying MDS and assessment of somatic UBA1 mutation.

## 1. Introduction

Recently, the concept of autoinflammatory disorders keep evolving and an increasing number of new monogenic diseases are identified but numerous autoinflammatory diseases are still to be characterized. Systemic autoinflammatory disorders (SAID) are unprovoked episodic or chronic sterile inflammation which are secondary to innate immune system dysregulation [1]. If well-characterized monogenic diseases such as familial Mediterranean fever are part of it, more than 50% patients presenting with clinical features resembling SAIDs still do not carry any of the know pathogenic mutations in autoinflammatory disease genes [2]. Some patients with SAID fit the criteria for polygenic forms of autoinflammatory diseases, such as adult-onset Still’s disease (AOSD); however, some of them do not. They are defined as undifferentiated systemic autoinflammatory disorders (USAID) [1,3].

Myelodysplastic syndrome (MDS) and chronic myelomonocytic leukemia (CMML) are part of a heterogeneous group of clonal hematopoietic stem cell diseases characterized by ineffective hematopoiesis, peripheral cytopenia with an increased risk of progression, to acute myeloid leukemia (AML) [4]. About 10 to 20% of MDS/CMML could be associated with various extra hematological features, e.g., neutrophilic dermatoses, systemic vasculitis, inflammatory arthritis, pseudo-Behçet’s disease, and relapsing polychondritis [5,6,7,8]. For years now, the state of autoinflammatory disorders associated with neoplasia reported in the literature were mainly associated with solid cancer but also MDS/CMML, myeloproliferative disorders, and lymphoma, and identified in the literature as “paraneoplastic AOSD” or “malignant associated AOSD” [8,9,10,11,12,13,14,15,16,17].

Recently, VEXAS (vacuoles, E1 enzyme, X-linked, autoinflammatory, somatic), a newly established X-linked disease associated with a somatic mutation in a gene encoding ubiquitin-like modifier activating enzyme 1 (*UBA1*) characterized by lipid vacuole inclusions in myeloid precursors of bone marrow examination, has been described [18]. These patients often develop hematologic malignancies, and especially MDS.

In this French nationwide study, our objective was to describe patients with MDS/CMML with unexplained recurrent fever and extra hematological signs, concordant or close to AOSD, and fulfilling the definition of USAID, their management, outcomes, overall survival, and the specific risk of progression to AML. We evaluated the prevalence of the newly described VEXAS syndrome in this group of patients and specifically reported their features.

## 2. Materials and Methods

### 2.1. Study Design

Data for patients with MDS or CMML and unexplained recurrent fever associated or not to extra hematological signs, concordant with USAID diagnosed between 2010 and June 2020 were retrospectively collected. All patients provided informed consent, and the study followed the Helsinki declaration and received the approval from the Institutional Review Board of Cochin Hospital (CLEP Decision N°: AAA-2021-08040). In agreement with the Committee for the Protection of Individuals—Regional Health Agency of Ile-de-France, given the retrospective nature of this French study, written informed consent from the patients was not required but the non-opposition of patients was necessary for inclusion. Three hundred and ninety patients were included in the MINHEMON registry across France for extra-hematological features associated to MDS/CMML. From this cohort, we selected patients which presented at least fever of unknown origin and elevated phase reactant. Physicians were also asked by Société Nationale Française de Médecine Interne” (SNFMI), “Club Rhumatisme et Inflammation” group (CRI), “Club Médecine INterne, HEMatologie et ONcologie” (MINHEMON) and the “Centre de référence des maladies auto-inflammatoires et des amyloses d’origine inflammatoire” CEREMAIA to report cases of patients with these features associated with MDS or CMML. Inclusion criteria were as follows: (1) MDS or CMML (WHO criteria); (2) AOSD with complete criteria (Yamaguchi and/or Fautrel criteria), or suspicion of AOSD (at least 3 from 5 criteria including fever) consistent with USAID; and (3) an established diagnosis of AOSD and MDS/CMML within the period of 5 years to avoid any fortuitous association. Exclusion criteria was defined as a diagnosis compatible with another autoimmune, infectious, drug-induced or systemic disease at the time of the inclusion. The diagnosis of suspected AOSD or USAID was made based on a consensus by two specialists (MD, AM) and a senior physician from the reference center of autoinflammatory diseases (SG). We compared our patients with a control group of MDS without extra hematological symptoms. Finally, we decided to screen the included patients for the newly described VEXAS to identify if it could be a new phenotype of this disease.

### 2.2. MDS, CMML

MDS and CMML were diagnosed based on peripheral blood and bone marrow examinations and classified according to the WHO 2016 criteria [19]. Patients were graded using the Revised International Prognostic Scoring System (IPSS-R) [20] for MDS: very low, low, intermediate, high, and very high risk groups; and the new CMML-specific Prognostic Scoring System (CPSS) for CMML: low, intermediate, and high. Karyotypes, the percentage of medullar blasts, and somatic mutations were assessed at the time of MDS diagnosis.

### 2.3. Genetic Analysis for VEXAS

Somatic *UBA1* mutation was explored by incorporating the targeted gene in a homemade 75-gene panel for the next generation sequencing (NGS) of circulating mononucleated cells with a 2% threshold of detection already used in previous work [21]. Only patients alive or dead with available DNA were tested for *UBA1.* Tested samples were collected in a single center except for one tested in his reference center. *UBA1* exon 3 sequences were determined by specific PCR followed by Sanger sequencing reaction using BigDye ™ Terminator (Applied Biosystems, Waltham, MA, USA) according to the manufacturer’s instructions and using the following primers: (forward) 5′-GTGGGTGGGAAAGTCTTTTGT-3′ and (reverse) 5′-TTACAGCTGCCGGGAGTAAAG-3′. A 3500xL-Dx Genetic Analyzer sequencing system was used, and sequences were analyzed with SeqScape software (Applied Biosystems). As MDS patients from the control group did not show any extra hematological features, they were not screened for *UBA1* mutation. *UBA1* mutated patients were identified and included in French VEXAS group cohort whom data could be used for other studies. The patients used in different studies are represented with an asterisk* in corresponding table.

### 2.4. USAID

Patient’s clinical files were screened for other autoinflammatory diagnostics. Symptoms of AOSD were specifically recorded and highlighted: fever, skin rash, arthritis or arthralgia, myalgia, pharyngitis or sore throat, nodes, or splenomegaly as well as these severe complications pericarditis, pleuritis, myocarditis, reactive hemophagocytic syndrome (RHS), disseminated intravascular coagulation (DIC), acute respiratory distress syndrome (ARDS), and interstitial lung disease (ILD).

Data from the laboratory test included complete blood count, liver enzymes, C-reactive protein (CRP), ferritin, and glycosylated ferritin levels.

USAID complete clinical and biological response was defined as a total disappearance of all clinical features present at baseline in exclusion of those possible reliable to MDS and/or CMML and a complete normalization of acute-phase reactants. Partial response was defined as an improvement of at least one the clinical sign without an increase of any other features and at least 50% improvement of acute-phase reactants. Patients treated with corticosteroids as first line were differentiated into two groups: high dosage (0.8 to 1 mg/kg/day of prednisone-equivalent) and low dosage. USAID diagnosis was considered concomitant with MDS/CMML or AML when the diagnosis of both diseases was made within ±3 months before or after MDS/CMML were diagnosed. The overall survival, the cause of death and the progression toward AML, were recorded, as well as the date of the last visit.

### 2.5. Control Group

The control group consisted of 104 patients from the “Groupe Francophone des Myelodysplasies” registry of MDS/CMML which were seen at least once by the internal medicine specialists (AM, OF). These patients did not exhibit any systemic inflammatory or autoimmune features and were selected without matching.

### 2.6. Ethics

All patients provided informed consent, and the study followed the Helsinki declaration.

### 2.7. Statistical Analysis

Descriptive data included medians and interquartile ranges (IQR) for continuous variables and frequencies (expressed in percentages) for categorical variables. To account for missing data, results were expressed as observed data (missing data were not replaced). The Chi-square or Fischer’s exact tests were used to compare categorical variables, and the Student’s *t*-test or Mann–Whitney U test were applied for the comparison of continuous variables. The significance of findings was determined by a *p*-value of 0.05 or less. Statistical analyses were performed using 2009 GraphPad software, San Diego, CA, USA v6.1.

## 3. Results

### 3.1. Characteristics of Patients

**Figure 1:**
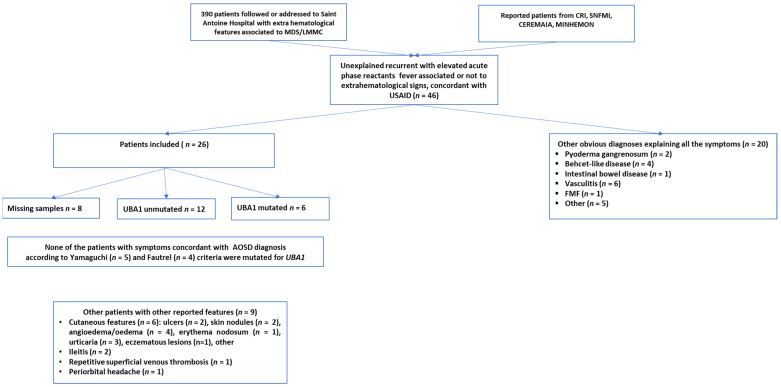
Flow-chart depicting patient selection. From the 46 patients screened, 26 were included. Six *UBA1* mutated patients identified from the included patients.

All 26 patients with USAID presented with recurrent unexplained fever and elevated acute phase reactants (Figure 1). The median age of appearance of USAID’s symptoms was 70.5 years (IQR: 64.3–78.9) with 76% being male (Table 1). Besides unexplained recurrent fever, clinical features of USAID included arthritis (*n* = 16, 62%) and skin rash (*n* = 11, 42%). Severe complications were noted at the time of diagnosis or during the follow-up period in nine patients (35%). These included RHS (*n* = 5), myopericarditis (*n* = 3), interstitial lung disease (*n* = 3), and ARDS (*n* = 1) (Table 1). Five patients among the initially identified USAID, presented symptoms concordant with a diagnosis of AOSD according to Yamaguchi (*n* = 5) and Fautrel (*n* = 4) criteria [22,23]. Nine patients with USAID, reported atypical signs for AOSD including other skin lesions (*n* = 7, 78%) such as ulcers, nodules, angioedema, erythema nodosum, urticaria, eczematous lesions; non-infectious ileitis (*n* = 2, 25%), recurrent superficial venous thrombosis (*n* = 1, 11%), and periorbital headache (*n* = 1, 11%).

MDS/CMML characteristics (WHO classification, cytogenetics, and prognostic scores) are shown in Table 2. The most frequent subtype was MDS with excess blasts (30.8% of the patients) (MDS EB-1: 11.5%, MDS EB-2: 19.2%), with a median R-IPSS of 3.7 (IQR: 2–5). NGS somatic mutations screening was available for 17 patients and showed TET2 (*n* = 5), ASXL1 (*n* = 4), IDH1 (*n* = 3), and KRAS (*n* = 3) mutations (Supplementary Table S1). The diagnosis of USAID co-occurred with preceded and appeared after respectively the diagnosis of MDS/CMML in eight (32%), twelve patients (48%), and five patients (24%). The median time between USAID and MDS/CMML diagnosis was one year (IQR: 0.2–2). Specific hematological treatments were initiated in seventeen cases (65%) within half a year (IQR: 0.2–1.2) after the diagnosis of MDS. First-line treatment mostly consisted of azacytidine (Supplementary Table S2).

Among the 26 patients included, 18 had samples available to be tested for *UBA1* mutation was tested in 18 patients and revealed six 33% positive cases, five out six were males (Figure 1 and Table 3). None of these patients progressed to AML during the 2.2 median years of follow up (ranges 1.2 to 5.6 years). Among these six individuals with VEXAS syndrome, none had a confirmed AOSD, including one with incomplete AOSD criteria sets and five remainders presenting additional unusual clinical features for AOSD. None had polychondritis, one patient hand interstitial pneumonia, one alveolitis, and five had cutaneous features distinct from a rash.

**Figure 2:**
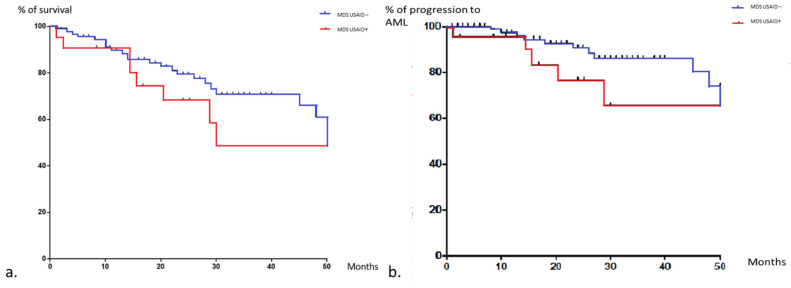
Kaplan–Meier curves of overall survival (**a**), progression to AML (**b**) in MDS patients with and without USAID. Data were censored at time of last visit or death. The response MDS control group is in blue and the one with autoinflammatory features is in red.

Compared to MDS/CMML controls without any inflammatory features (*n* = 104), USAID-associated MDS/CMML patients were younger (71.4 (IQR: 36–85) vs. 78 years (IQR: 42–92); *p* = 0.0005), without any other statistically significant differences for demography, MDS/CMML subtype, survival. Even if the rates of deaths were higher in the study group (46% vs. 25%; *p* = 0.02), the overall survival and the time to progression to AML were not significantly different between the groups (Figure 2).

### 3.2. Treatment and Outcomes

Twenty-two patients (85%) received specific treatment for USAID. Corticosteroids were used as a first-line treatment in 19 cases (86%) alone (*n* = 17) or combined with anakinra or infliximab in the other 2 cases. A complete clinical and biological response to first-line treatment was observed in 14 cases (64%) but 5 cases of secondary steroid dependency were described. Thirteen patients (59%) required a second-line treatment because of a resistance to steroids, primary inefficiency, or relapse, with a response in eight (73%) patients. Targeted biological therapy alone or in association with corticosteroids enabled either a partial or complete response in 9 out of 11 patients (anakinra *n* = 8/9, tocilizumab *n* = 0/1, and infliximab *n* = 1/1). Azacytidine used for MDS or steroid dependent autoinflammatory disease (*n* = 12) resulted in a complete or partial response of systemic features in 10 (83%) cases. Seven patients (26%) progressed to AML and 12 died (46%), all except one in the VEXAS negative group during the follow-up (Supplementary Table S2 and Figure S1).

Among the six patients with VEXAS, four responded temporarily and/or partially to corticosteroids: relapses and steroid dependency were observed in all cases. Anakinra was used in five cases with a complete or partial response in four cases. Tocilizumab was ineffective in one remaining case. When available, azacytidine led to a response on systemic features in all 3 cases.

## 4. Discussion

Patients with MDS/CMML can develop USAID with various extra hematological features including pattern suggestive of AOSD. This is the first multidisciplinary series (involving both rheumatologists, hematologists, and clinical immunologists) reporting on USAID associated with MDS/CMML features and the prevalence of VEXAS in such cohort(s). The results of the present study have several important implications. In the case of MDS/CMML with recurrent and unexplained fever, our cohort, although limited, shows that VEXAS syndrome represents 33% (6/18) of cases, these inflammatory situations remaining rare. Moreover, over a median follow-up of 26 month, none of the 6 cases of MDS/CMML VEXAS + progressed to AML, versus 23% of MDS/CMML with USAID and 40% of classic MDS/CMML without inflammatory manifestations. At last, although the numbers are small therefore caution must remain prevalent, there could be a potential interest of biological therapies or even azacytidine when *UBA1* is mutated.

Patients with MDS and unexplained recurrent fever should be first tested for the *UBA1* mutation. The recent work by Beck et al. which identified VEXAS, implying that somatic mutations may be a more frequent cause of human disease than previously recognized [18,24]. A third of our patients screened for *UBA1* were positive for this mutation (including one woman). As a cohort of comparison, in relapsing chondritis, a frequently associated clinical picture usually described with VEXAS, only 7.6% had UBA1 mutations [25]. USAID appeared as a new pattern of VEXAS. Interestingly, 50% of our VEXAS patients experienced RHS. In the future, the order of *UBA1* screening could be debated: integrated into the first-line genetic exploration of MDS as well as the MDS/SMP NGS panel or rather screened in the case of MDS/CMML with systemic features in particular USAID.

In contrast to other dysimmune manifestations with a near equal frequency distribution before, simultaneously, and after the diagnosis of MDS/CMML (USAID and systemic features of VEXAS tend to precede the diagnosis of hematological diseases) [9]; hence, the importance of an early identification of warning signs to carefully consider an underlying neoplasia, in particular hematological malignancy. Nearly 20% of the patients matched with the criteria concordant with AOSD, but some atypia should raise the physicians’ attention in patients with newly-diagnosed AOSD, with some features such as late-onset disease [26], male sex [27] as well as cytopenia in particular the absence of leukocytosis (a hallmark feature of AOSD), and macrocytosis and monocytosis [9,11]. In a recent Dutch case series of VEXAS, among patients with unclassified autoinflammation, two among the twelve retrospectively identified were initially suspected with AOSD [28]. Thus, elderly patients with late AOSD diagnosis or suspicion as described in some series [29,30] could also benefit from *UBA1* mutation screening.

As described previously for systemic inflammatory and autoimmune disorders associated with MDS/CMML, MDS EB, and MDS MLD were the most frequently associated subtypes in USAID patients not *UBA1* mutated [6,31]. No association with a specific karyotype was identified but interestingly *TET2* and *IDH* mutation already associated with systemic inflammatory and auto immune disease and T cell dysregulation in a recent work [32] were also frequently found in our cohort. Two out of the six VEXAS patients presented *DNMT3A* also described in other VEXAS patients [18,28,33,34], but significance is yet unclear. None of the patients with VEXAS had CMML, which is consistent with the recent report published by Zhao et al. [35]. The prognosis of MDS-associated autoimmune disorders remains unclear, as depending on the types of autoimmune disease in each cohort, the impact on survival could be different [7] but we must notice that in our group, despite a high rate of transformation (more than 25%), the twelve death: only one was *UBA1* mutated and none of the VEXAS patients transformed into AML.

The treatment of symptoms of patients with malignant associated features concordant or close to AOSD is challenging. Corticosteroids response was similar to AOSD’s but with a high percentage of steroid dependency or secondary failure [27]. Methotrexate, usually used for its steroid-sparing effect, was rarely applied, presumably due to the risk of cytopenia, explaining the frequent use of biologic agents. The preferential utilization of interleukin-1R antagonists rather than TNF-a antagonists usually used in articular rheumatism is justified by the dominance of the systemic character of the disease with frequent spikes in fever and seemed efficient. Most treatments were only transiently effective, but hematological treatment allowed control of the USAID symptoms for 11 patients in our cohort. As in autoimmune and inflammatory disorders, azacytidine, appears to have a beneficial effect on these systemic manifestations [36,37]. Similarly, in a recent retrospective, Bourbon et al.’s study of the hypomethylating agent and signaling inhibitors seemed to achieve interesting results in VEXAS syndrome patients [38]. The occurrence of USAID or AOSD compatible features over the course of myelodysplastic diseases may prompt a hematological treatment, especially in its refractory form, despite the absence of a direct hematological indication. Thus, azacytidine could be a therapeutical hypothesis for USAID associated with MDS as well as it could be for *UBA1* mutated patients.

Our study has several important limitations. First, the retrospective design used here carries an inherited risk of bias and missing data. Second, despite the scarcity of the described clinical syndrome, a small sample size limits the generalizability of the findings reported. Herein we chose to select only patient with occurrence of USAID/AOSD suspicion and MDS/CMML within the period of 5 years to avoid any fortuitous association. Further studies should aim at improving study designs with a prospective nature, larger sample size, and detailed comprehensive information collection.

## 5. Conclusions

Systemic form of VEXAS syndrome can mimic AOSD, especially in the elderly. USAID symptoms including a pattern suggestive of AOSD associated with MDS/CMML was identified in 33% cases with VEXAS syndrome, thereby further expanding the spectrum of this new syndrome. *UBA1* should be sequenced in this population, especially in case of macrocytic associated anemia and chronically elevated CRP among old males, but not exclusively. Although the optimal management of UBA1/MDS overlap patients remains to be determined in larger scale cohorts, biological therapies and/or hypomethylating agents seem promising in this situation.

## Key messages:

- Patients with MDS/CMML can develop a recurrent fever with various extra hematological features suggestive of USAID;
- Pseudo-Still disease or USAID associated with a myelodysplastic syndrome are a new phenotype of VEXAS syndrome;
- *UBA1* mutation should be screened in case of such association.

## Figures and Tables

**Table 1:** Baseline characteristics of USAID including pattern suggestive of AOSD.

	Patients (*n* = 26)	No VEXAS (*n* = 18)	VEXAS (*n* = 6)
Epidemiology			
Male-to-female ratio	3.16	2.8	4
Age at 1st signs (years) (range)	70.5 (64.3–78.9)	71 (65.5–79.3)	64.3 (63.6–68.2)
Time between USAID and MDS (years) (range)	1.0 (0.2–2)	0.9 (1.8–0.2)	1.2 (1.1–3)
Medical history (%)			
Personal or familial history of autoimmunity	2 (9)	1 (6)	1 (25)
Symptoms (%)			
Fever	26 (100)	18(100)	6 (100)
Skin rash	11 (42)	6 (33)	5 (83)
Arthritis/arthralgia	16 (62)	14 (78)	2 (33)
Pharyngitis/sore throat	5 (19)	1 (22)	1 (17)
Myalgia	5 (19)	3 (17)	2 (33)
Lymph nodes or splenomegaly	12 (46)	8 (44)	4 (67)
Blood test results (%)			
Leukocytosis > 10,000/mm^3^	10 (38)	9 (50)	1 (17)
Granulocytes ≥ 80%	7 (26)	6 (67)	1 (17)
CRP >30 mg/L	26 (100)	18 (100)	6 (100)
Elevated liver enzymes	9 (35)	7 (39)	2 (67)
High ferritin	19/20 (95)	15 (94)	4 (100)
>2000 µg/L	14/20 (70)	13 (81)	1 (25)
Glycosylated ferritin < 20%	4/11 (36)	3 (30)	1 (50)
Severe complications			
Pericarditis	3 (12)	3 (18)	0 (0)
Myocarditis	0 (0)	0 (0)	0 (0)
DIC	0 (0)	0 (0)	0 (0)
RHS	5 (19)	2 (11)	3 (50)
ARDS	1 (4)	1 (4)	0 (0)
ILD, alveolitis	3 (12)	1 (4)	2 (67)
Pleurisy	6 (23)	6 (33)	0 (0)

AOSD, adult-onset Still’s disease; ARDS, acute respiratory distress syndrome; CRP, C-reactive protein; DIC, disseminated intravascular syndrome; ILD, interstitial lung disease; MDS, myelodysplastic syndrome; RHS, reactive hemophagocytic syndrome.

**Table 2:** Main features of MDS/CMML patients with USAID.

	MDS-Related USAID *n* = 26 (%)	MDS/CMML Controls *n* = 104 (%)
Mean age at diagnosis (range)	71.4 (36–85)	78 (42–92) *
Male	19 (76)	79 (76)
Myelodysplastic syndrome (MDS)	23 (89)	83 (80)
MDS with single lineage dysplasia	3 (12)	14 (13)
MDS with ring sideroblasts (MDS-RS)	2 (8)	6 (6)
MDS with multilineage dysplasia	5 (19)	33 (32)
MDS with excess blasts (EB)	8 (31)	14 (13)
MDS with isolated del(5q)	0	4 (4)
MDS, unclassifiable/missing data	5 (19)	7 (7)
Chronic myelomonocytic leukemia (CMML)	3 (12)	21 (20)
Acute myeloid leukemia (AML) (progressed to)	7 (27)	11 (10)
IPPS-R (IQR)	3.7 (2–5)	2.5 (0–8)
Deaths	12 (46)	26 (25) *
Follow-up (range)	16.8 (0–104)	21.5 (0–86)

* *p* value < 0.05.

**Table 3:** Main features of the patients with *UBA1* mutations (VEXAS syndrome).

Patient	Sex	Type MDS	Age at Diagnosis of MDS	Characteristics of MDS	Age at USAID Onset	Signs of USAID	Other Symptoms	Evolution
Patient #1 *	M	MDS UL	59.8	IPPS = NS	58.8	Fever, rash, cytolysis, high ferritin, RHS	Recurrent superficial veinous thrombosis, cutaneous atypia	Azacytidine for MDS just begun
				NGS: *DNMT3A, BRCA2*, and *ZRSR2* mutated Bone marrow: vacuoles in myeloid precursor cells				No response to CTC high dose rapidly completed with cyclosporine permitting, PR
				Del 6q Blasts: NS				Alive under azacytidine, CTC 20 mg and cyclosporine
Patient #2 *	M	MDS EB1	64.5	IPPS = 4	63.3	Fever, arthralgia, leukocytosis, lymphadenopathy, splenomegaly, granulocytes, cytolysis		CR under azacytidine for MDS
				NGS: Normal			Periorbital headache, skin nodules, edema, diarrhea, abdominal pain	Multiple lines of treatments: response and relapses to high-dose CTC and anakinra; no response to infliximab, tocilizumab, IV Ig, cyclosporine
				Blasts: 7%				Alive and disappearance of systemic features under azacytidine and CTC 20 mg/day
Patient #3 *	M	Unclassifiable MDS	73.5	IPPS-R: 2	73.5	Fever, rash, lymphadenopathy, splenomegaly, high ferritin, RHS	Recurrent face edema, urticaria, skin nodules and eczematous skin lesions	PR with azacytidine for MDS
				NGS: *DNMT3A* mutated 31.7%				No response to high-dose CTC and anakinra
				Blasts: 0%				Alive, PR and systemic symptoms with azacytidine
Patient #4	F	Unclassifiable MDS	64.3	IPPS-R: NS	NS	Fever, rash, RHS		Unknown response with hydroxyurea
				Del X Blasts: NS			Interstitial pneumonia, diarrhea	CR with high-dose CTC, steroid dependence; CR with anakinra and CTC
								Alive at last visit
Patient #5 *	M	MDS MLD	69.4	IPPS-R: 2 NGS: *TET2* mutated 3%	65.8	Fever, rash, arthritis, sore throat, lymphadenopathy, splenomegaly, high ferritin, myalgia	Pustular eruption and other cutaneous atypia	CR with azacytidine; failure with methotrexate; leflunomide stopped early due to cytopenia; CR (with few relapses) with CTC and anakinra
Patient #6	M	Unclassifiable MDS	NS	IPPS-R: NS NGS: normal	69	Fever, rash, high ferritin, myalgia	Oedema, angioedema chronic urticaria, alveolitis	No hematological treatment CR under high dose CTC but CTC dependency, CR under anakinra and CTC stopped for neutropenia Death at 73 years from sepsis and cardiac failure and anakinra
Median (yrs) (IQ 25;75) *n* = (%)	M/F = 5:1	Follow up 2.2 (1.5; 3.3)	64.5 (64.3; 69.4)			Fever (*n* = 6, 100%), skin rash (*n* = 5, 83%), arthralgia arthritis (*n* = 2, 33%), lymph nodes/splenomegaly *n* = 3 (50), sore throat (*n* = 1, 17%), RHS *n* = 3 (50), leukocytosis (*n* = 1, 17%), granulocytosis (*n* = 1, 17%), cytolysis (*n* = 2, 33%), high ferritin (*n* = 4, 67%)		

CR, complete remission; CTC, corticosteroids; Ig, immunoglobulin; IPPS-R, Revised International Prognostic Scoring System; IV, intravenous; MDS, myelodysplastic syndrome; MDS EB1, MDS with excess of blasts < 5%; MDS: myelodysplastic syndrome; NGS, next-generation sequencing; NS, not significant; PR, partial response; RHS, reactive hemophagocytic syndrome; MDS SLD, MDS with single lineage dysplasia; MDS UL. Patients marked with an * were identified and included in French VEXAS group cohort and could have been used in other published studies.

## Data Availability

Data are available on request from the corresponding author.

## Supplementary Materials

The following are available online at https://www.mdpi.com/article/10.3390/jcm10235586/s1, Figure S1: Different lines of USAID treatments and treatment responses. Twenty-two patient (85%) received specific treatment for USAID and 14 (64%) experienced complete response. Fifty-nine percent of the patient needed a second line treatment and 54% a third line, Table S1: Main features of MDS/CMML patients with USAID, Table S2: USAID including pattern suggestive of AOSD natural history and therapeutical management.

## Abbreviations

A	Arthritis/arthralgia
AML	Acute myeloid leukemia
ANA	Antinuclear antibody
AOSD	Adult-onset Still’s disease
ARDS	Acute respiratory distress syndrome
C	Cytolysis
CBC	Complete blood count
CC	Corticosteroids
CMML	Chronic myelomonocytic leukemia
CPSS	CMML-specific prognostic scoring system
CR	Complete remission
CRP	C-reactive protein
DIC	Disseminated intravascular coagulation
F	Female
F	Fever
Fr	High ferritin
G	Low glycosylated ferritin
IL	Interleukin
ILD	interstitial lung disease
L	Lymph nodes and splenomegaly
M	Male
M/F	Male-to-female ratio
MAS	Macrophage activation syndrome
MDS	Myelodysplastic syndrome
MDS MLD	MDS with multilineage dysplasia
MDS SLD	MDS with single lineage dysplasia
MDS-EB1	MDS with excess of blasts <5%
MDS-EB2	MDS with excess of blasts >5%
MDS-RS	MDS with ring sideroblasts
MM	Myelodysplastic malignancy
MTX	Methotrexate
PNN	Polymorphonuclear neutrophils >80%
PET scan	Positron emission tomography scan
PR	Partial remission
RF	Rheumatoid factor
RHS	Reactive hemophagocytic syndrome
R-IPPS	Revised International Prognostic Scoring System
SAID	Systemic autoinflammatory disorder
S	Skin rash
T	Sore throat
TNF	Tumor necrosis factor
UBA1	Ubiquitin-like modifier activating enzyme 1
USAID	Undifferentiated systemic autoinflammatory disorder
VEXAS	Vacuoles, E1 enzyme, X-linked, Autoinflammatory, Somatic syndrome
W	Leukocytosis
Yrs	Years
