# Supplementary material for: USAID Associated with Myeloid Neoplasm and VEXAS Syndrome: Two Differential Diagnoses of Suspected Adult Onset Still’s Disease in Elderly Patients
Source: J Clin Med. 2021 Nov 27;10(23):5586. doi: 10.3390/jcm10235586 (PMC8658409; doi:10.3390/jcm10235586)
Supplement: Supplementary file 1 [file jcm-10-05586-s001.zip › jcm-1425139-supplementary.pdf]

Supplementary Table S1. Main features of MDS/CMML patients with USAID

**Myelodysplastic syndromes (MDS)**		n=26
MDS with single lineage dysplasia (MDS SLD)		3
MDS with ring sideroblasts (MDS-RS)	MDS-RS and single lineage	0
	MDS-RS and multilineage dysplasia	2
MDS with multilineage dysplasia (MDS MLD)		5
MDS with excess blasts (EB)	EB-1	3
	EB-2	5
MDS with isolated del(5q)		0
MDS, unclassifiable		5
**Karyotype**		n=25
MDS	Very good	0 (0)
	Good	12 (50)
	Intermediate	6 (29.2)
	Poor/very poor	5 (20,8)
Chronic myelomonocytic leukemia (CMML)	Low	1 (50)
	Intermediate	1 (50)
	High	0 (0)
**NGS**		n=17
TET2		5 (29)
ASXL1		4 (24)
IDH1		3 (18)
KRAS		3 (18)
Negative		5 (29)
**Bone marrow blasts (%)**	MDS	4%
**Prognostic score**		n=19
IPSS-R	Very low (≤1,5)	2 (11.8)
	Low (1,5< IPSS-R≤3)	6 (35.3)
	Intermediate (3< IPSS-R≤4,5)	3 (17.7)
	High (4,5<IPSS-R≤6)	3 (17.7)
	Very High (>6)	3 (17.7)
CPSS	Low	1 (50)
	Intermediate 2	1 (50)

Table S2. AOSD and USAID natural history and therapeutical management

	**All patients (n=26)**
Concomitant diagnosis	8 (32)
AOSD/USAID preceded myeloid neoplasm	12 (48)
Myeloid neoplasm preceded AOSD/USAID	5 (20)
AOSD/USAID outcomes to hematological treatment (azacytidine (n=12), allograft (n=1), cytarabine (n=1), hydroxyurea (n=1), IDH1 inhibitor (n=1)	
Response in both diseases	12 (70)
No response in both diseases	1 (6)
Response in hematological disease only	2 (12)
**USAID response to the first-line treatment**	18 (81)
**MDS/CMML response to the first-line treatment**	7 (70)
**Response to USAID treatment**	
**Treated with corticosteroids**	22 (100)
Complete remission	15 (75)
Partial remission	2 (10)
**Treated with anakinra**	9 (41)
Complete remission	7 (78)
Partial remission	1 (11)

Figure S1. Different lines of USAID treatments and treatment responses

First-line treatment n=22
Complete response n=14 (64%) including n=11 among high CCs dose
Partial response n= 4 (18%) including n=2 among high CCs dose
No response n=4 (18%) including n=3 among high CCs dose
Early stop for side effect n=1 (5)
Missing data n= 0
Steroid dependence n=5 (23%)

→

Steroids n=19 (86%) alone n=17, combined with other treatment n=2, high dose n=16, low dose n=3
Biologics n=3 (14%) (Anakinra n=2, Infliximab n=1)
Methotrexate n=1 (5%)
NSAID n=1 (5%)

Second-line treatment n=13
Complete response n=7 (64%) including n=6 under anakinra
Partial response n=1 (9%)
No response n= 3 (27%)
Early stop for side effect n=3 (27%)
Missing data n=2 (18%)
Steroid dependence n=1 (9%)

→

Steroids n=10 (77%) combined with anakinra n=7
Biologics n=7 (54%) (Anakinra)
Methotrexate n=3 (23%)
Hydroxychloroquine n=1 (8%)
Leflunomide n=1 (8%)
Ciclosporine n=1 (8%)

Third-line treatment n= 7
Complete response n=2 (33%) including n=1 under anakinra
Partial response n=2 (33%)
No response n=2 (33%) including n=1 under tocilizumab
Early stop for side effect n=1 (17%)
Missing data n=1 (17%)
Steroid dependence n=1 (17%)

→

Steroids n=6 (86%) combied with anakinra n=1
Biologics n=2 (28%) (Anakinra n=1 and Tocilizumab n=1)
Hydroxychloroquine n=2 (28%)
Colchicine n=1 (14%)
